# Pyrroloquinoline quinone regulates the redox status *in vitro* and *in vivo* of weaned pigs via the Nrf2/HO-1 pathway

**DOI:** 10.1186/s40104-021-00595-x

**Published:** 2021-06-18

**Authors:** Caiyun Huang, Zijuan Fan, Dandan Han, Lee J. Johnston, Xi Ma, Fenglai Wang

**Affiliations:** 1 State Key Lab of Animal Nutrition, College of Animal Science & Technology, China Agricultural University, Beijing, 100193 China; 2 Swine Nutrition and Production, West Central Research and Outreach Center, University of Minnesota, Morris, MN USA; 3 Department of Internal Medicine/Department of Biochemistry, University of Texas Southwestern Medical Center, Dallas, TX USA

**Keywords:** H_2_O_2_, IPEC-J2, Nrf2, Oxidative stress, Pyrroloquinoline quinone, Weaned pig

## Abstract

**Background:**

Oxidative stress is a main cause of piglet gut damage and diarrhea. Pyrroloquinoline quinone (PQQ), is a novel redox cofactor with antioxidant properties. However, the effect and mechanism that PQQ supplementation decreases oxidative injury in weaned pigs is not understood. Therefore, the aim of this study is to confirm the effect of PQQ on regulating redox status in weaned pigs and the mechanism for antioxidant function by porcine intestinal epithelial cell line (IPEC-J2) challenged with H_2_O_2_.

**Results:**

Experiment 1, 144 Duroc × Landrace × Yorkshire pigs (weaned at 28 d) were allocated to four groups: received a basal diet (control) and diets supplemented with 0.15%, 0.30% and 0.45% PQQ, respectively. On d 28, growth performance, diarrhea incidence and redox factors were measured. Experiment 2, IPEC-J2 were treated with or without PQQ in the presence or absence of H_2_O_2_ for indicated time points. Experiment 3, IPEC-J2 were transfected with or without Nrf2 siRNA, then treated according to Experiment 2. The cell viability, redox factors, protein of tight junctions and Nrf2 pathway were determined.

*In vivo*, PQQ supplementation demonstrated dose-related improvements in average daily gain, and gain to feed ratio (Linear *P* < 0.05). During d 0–28, compared to controls, 0.45% PQQ supplementation for pigs decreased diarrhea incidence and MDA content in liver and jejunum, and increased concentration of SOD in liver; 0.3% PQQ supplementation decreased ileal and liver MDA concentration; and 0.15% PQQ supplementation decreased ileal MDA concentration (*P* < 0.05). *In vitro*, compared to cells cultured with H_2_O_2_, pre-treatment with PQQ increased cell viability, tight junction proteins expression including ZO-1, ZO-2, Occludin and Claudin-1; and decreased ROS concentration and level of Caspase-3 (*P* < 0.05); as well as upregulated the ratio of Bcl-2 to Bax and protein expression of nuclear Nrf2, HO-1. Notably, Nrf2 knockdown by transfection with Nrf2 siRNA largely abrogated the positive effects of PQQ pretreatment on H_2_O_2_-induced intracellular changes.

**Conclusions:**

PQQ administration attenuated oxidative stress in weaned pigs which is associated with activation of Nrf2/HO-1 pathway.

**Supplementary Information:**

The online version contains supplementary material available at 10.1186/s40104-021-00595-x.

## Introduction

Oxidative stress leads to many diseases, such as sepsis and enteritis, in young animals [1, 2]. Early-weaned pigs suffer oxidative challenges that produce large amounts of reactive oxygen species (ROS) [3]. Continuous accumulation of ROS induces an imbalance in the Bax/Bcl-2 protein ratio and increases the caspase-3 level, which leads to cell death [4, 5]. As a result of cell death, the organizational structure of the gut, specifically tight junctions, is damaged, resulting in diarrhea and growth retardation [3, 6, 7]. Zinc oxide and many antibiotics have historically been added to diets for weaned pigs as a nutritional intervention to mediate oxidative stress induced by weaning. However, undesirable side effects, such as heavy metal pollution and bacterial antibiotic resistance, have arisen [8]. Thus, alternatives that ameliorate oxidative challenges without damaging the environment must be discovered.

Pyrroloquinoline quinone (PQQ) is a novel redox cofactor of microbial quinoprotein enzymes [9, 10] that has been proven to enhance growth and stress tolerance [11, 12]. Oral administration of PQQ was found to reduce oxidative injury [13] and inhibit programmed cell death [14] in rat cardiac myocytes. PQQ was also found to protect the liver against damage by regulating oxidative responses in hens [15] and mice [16]. In addition, dietary supplementation with pyrroloquinoline quinone disodium (PQQ•Na_2_) in gestating and lactating rats improved the intestinal barrier functions of their offspring [17]. In our previous study, dietary PQQ supplementation was found to regulate redox status and promote gut health in weaned pigs [18], but the mechanism underlying this response was unclear.

NF-E2-related factor 2 (Nrf2) is a key cellular sensor of oxidative factors such as ROS [19]. Normally, the inactive Nrf2-Keap-1 complex exists in the cytoplasm. However, after stimulation, Nrf2 dissociates from this complex to translocate into the nucleus, where it induces the expression of genes encoding various antioxidant molecules, such as heme oxygenase-1 (HO-1) [20]. Activation of the Nrf2 signaling pathway plays a protective role in pigs under weaning stress [21]. Notably, PQQ was found to increase the expression of Nrf2 in human renal tubular epithelial cells to prevent oxidative injury in a high-glucose environment [22]. However, it is currently unclear whether PQQ affects the activity of the Nrf2/HO-1 pathway to reduce oxidative stress in weaned pigs.

We hypothesize that PQQ supplementation can regulate whole-body redox status via the Nrf2/HO-1 signaling pathway. In the present study, we used weaned pigs and IPEC-J2 cells to evaluate the effect of dietary PQQ supplementation on the antioxidant defense capacity in postweaning pigs.

## Materials and methods

### Animals and diets

All experimental protocols used in the present study were approved by the Animal Care and Use Committee of China Agricultural University (AW30129102–3) and performed in accordance with the guidelines of the National Research Council’s Guide for the Care and Use of Laboratory Animals. Crossbred pigs (Duroc × Landrace × Yorkshire, *n* = 144, initial weight 7.58 ± 1.67 kg, weaned at d 28, half barrows and half gilts) were allocated randomly to one of four dietary treatment groups: a group received a corn-soybean meal-based diet (as a control) and three groups received experimental diets supplemented with 0.15%, 0.30%, and 0.45% PQQ•Na_2_ (Supplemental Tables 1 and 2). The basal diet was formulated according to ﻿the guidelines of the National Research Council [23]. For each treatment, we used six replicates (pens) with six pigs per pen (50% barrows and 50% gilts). Pigs were housed in pens (1.8m × 1.2m) that contained a nipple drinker and a four-hole self-feeder to provide ad libitum access to water and feed. The room was equipped with air conditioning and ventilation, and the temperature was 27 ± 1 °C. PQQ•Na_2_ (purity, ≥ 98%; Changmao Biochemical Engineering Co. Ltd., Changzhou, China) was diluted with corn starch to a concentration of 1 g/kg and was blended into the basal diet to achieve the desired treatment concentration; the concentration of PQQ in the basal diet was less than 0.01 mg/kg [18].

### Growth performance and sample collection

During the 28-day feeding trial, the diarrhea incidence of pigs was recorded by one person and was based on the following scale: 1 = well-formed feces, 2 = unformed feces, 3 = diarrhea [24]. The diarrhea incidence for pigs in a pen was calculated as follows: [(number of pigs with diarrhea × number of days of diarrhea)/(total number of pigs × number of days in the experiment)] × 100 [25].

On d 14 and d 28 of the experiment, pigs were weighed to calculate the average daily gain (ADG), and feed disappearance was used to calculate the average daily feed intake (ADFI) and gain-to-feed ratio (G:F) on a pen basis.

At the end of the feeding trial, blood was collected from the anterior vena cava of one pig per pen (total 24 pigs) after a 12-h fast. Harvested serum was stored at − 20 °C. The pigs from which blood samples were obtained were slaughtered for collection of intestinal, hepatic and cardiac tissue samples, which were stored at − 80 °C for further analysis.

### Histological examination of the intestinal morphology

The villus height (VH), crypt depth (CD) and villus height/crypt depth ratio (VCR) of three intestinal segments were analyzed based on methods used in our previous study [26]. In brief, one segment each of the mid-duodenum, mid-jejunum and mid-ileum were fixed with 4% neutral buffered paraformaldehyde for 24 h and were then dehydrated, embedded in paraffin, cut at a thickness of 4 μm, and stained with hematoxylin and eosin. Images were acquired with a microscope (Olympus, BX-51, Japan) and analyzed using an Image-Pro Plus (Media Cybernetics, USA). Six slides with two separate segments per slide were prepared for each sample, resulting in over 20 well-oriented villi and crypts for measurement.

### Redox factor expression

The activity of glutathione peroxidase (GSH-Px), superoxide dismutase (SOD), malondialdehyde (MDA) and catalase (CAT) in serum, liver, heart, and intestine samples and IPEC-J2 cells were detected using ELISAs according to the manufacturer’s instructions (Nanjing Jiancheng Bioengineering Institute, Nanjing, China). The absorbances were measured at 412 nm for GSH-Px, 550 nm for SOD, 532 nm for MDA, and 405 nm for CAT. The minimal detection thresholds were 20 U/mL for GSH-Px, 5 U/mL for SOD, 0.5 nmol/mL for MDA and 0.2 U/mL for CAT. For each assay, the intra-assay coefficient of variation (CV) was < 5%, and the inter-assay CV was < 6%. Six samples per tissue were tested for each treatment group, and each sample was assayed in triplicate.

### Cell culture

The IPEC-J2 porcine intestinal epithelial cell line was kindly provided by Dr. Guoyao Wu (Texas A&M University, College Station, TX, USA). Cells were cultured as described previously [27]_._ In brief, cells were cultured in DMEM/F12 medium (Thermo, Waltham, MA) containing 10% (vol/vol) fetal bovine serum (FBS; Gibco, Carlsbad, CA), 1% penicillin (10,000 U/mL)/streptomycin (10,000 g/mL; Gibco) and maintained in an atmosphere of 95% humidity with 5% CO_2_ at 37 °C.

### Cell viability

IPEC-J2 cells were cultured in 96-well plates. After reaching 90% confluence, cells were starved for 6 h. Hydrogen peroxide was added to cells for 2, 6, 18, and 24 h at concentrations of 0, 200 μmol/L, 500 μmol/L, 800 μmol/L, 1 mmol/L, 1.5 mmol/L, and 2 mmol/L. Additionally, PQQ•Na_2_ was dissolved in PBS and incubated with cells at concentrations of 0, 1 nmol/L, 10 nmol/L, 100 nmol/L, 1 μmol/L, 10 μmol/L, and 100 μmol/L for 6, 18, 36, and 48 h. For analysis of cell viability, cells were incubated with Cell Counting Kit-8 (CCK-8) solution (CK04, Dojindo, Kumamoto, Japan), the absorbances were measured at 450 nm in a microplate reader (Thermo Fisher Scientific, Grand Island, NY), and absorbance values were normalized to those of the control (CTRL) group.

### RNA interference and transfection of IPEC-J2 cells

The double-stranded specific small interfering RNA (siRNA) targeting Nrf2 was synthesized by RiboBio Co., Ltd. (Guangdong, China). The primer sequences were as follows: 5′-GCCCAUUGAUCUCUCUCAUTT-3′ and 5′-AUCACACACAUGGGCTT-3′. IPEC-J2 cells were incubated in six-well plates. After reaching 70% confluence, cells were transfected with 10 nmol/L, 50 nmol/L, or 100 nmol/L Nrf2 siRNA or negative control (NC) siRNA using Lipofectamine 3000 reagent (Invitrogen, Carlsbad, CA, USA) in Opti-MEM according to the product manuals.

### Western blot analysis

IPEC-J2 cells were transfected with or without 100 nmol/L Nrf2 siRNA and were then cultured with 10 nmol/L PQQ•Na_2_ for 6 h before replacement with medium containing 10 nmol/L PQQ•Na_2_ and 200 μmol/L H_2_O_2_ for another 2 h. Cells were harvested to analyze protein abundance and the methods described in our previous study [28]. In brief, cells were lysed in RIPA buffer containing Halt protease inhibitor cocktail (Thermo Fisher Scientific, Rockford, IL) for protein extraction. Nucleocytoplasmic fractionation was performed using NE-PER™ Nuclear and Cytoplasmic Extraction Reagents (78,833, Thermo Fisher Scientific). Proteins (30 μg) were separated on SDS-PAGE gels and transferred to polyvinylidene difluoride membranes (Millipore). After being blocked for 1 h, membranes were incubated with primary and secondary antibodies. The information of primary antibodies were listed in Supplemental Table 3. Band densities were detected and quantified with an Odyssey Clx system (Gene Company Limited, Hong Kong, China) and ImageJ software, respectively. All expression levels were normalized to those of Tubulin, PCNA or β-actin, as appropriate.

### Quantitative real-time PCR assay

Cells were cultured as described for western blot analysis. Quantitative real-time PCR was performed as previously described to analyze gene expression [29]. In brief, total RNA was extracted using TRIzol reagent (TaKaRa Bio Inc., Japan) according to the manufacturer’s instructions. cDNA was reverse transcribed from RNA with a reagent kit (TaKaRa Bio Inc., Japan). Quantitative real-time PCR was conducted in an ABI 7500 Real-Time PCR system (Applied Biosystems, Foster City, CA, USA). The primer sequences used in this study are listed in Supplemental Table 4. Each gene was analyzed in triplicate, and *β*-actin was used as the reference gene. The relative mRNA expression levels of the target genes were determined by the 2^-ΔΔCT^ method [30].

### Immunofluorescence staining

IPEC-J2 cells were cultured in a 24-well plate. Cells were treated as described for western blot analysis. The methods used for immunofluorescence staining of cells were described previously [31] and were followed with slight modifications. In brief, after 30 min of fixation with 4% paraformaldehyde solution, cells were permeabilized with 0.2% Triton X-100 for 10 min and blocked with 5% BSA for 1 h. Cells were incubated with a rabbit anti-Nrf2 antibody overnight at 4 °C and were then incubated with an Alexa Fluor 594-conjugated secondary antibody (ZF-0513, ZSGB-BIO) for 1 h. Subsequently, cell nuclei were stained with DAPI (Alexa Fluor® 555; ab150078; Abcam) for 10 min and imaged immediately using an Olympus fluorescence microscope (Tokyo, Japan). Each treatment group was analyzed in triplicate.

### Statistical analysis

﻿﻿Normal distribution was validated for diarrhea incidence data by QQ plot, then, the data was evaluated by the GLIMMIX procedure. Other data from the animal experiment in this study were analyzed using the MIXED procedure and polynomial contrasts (SAS, version 9.2). Initial body weight and sex were treated as random effects, and the PQQ supplementation concentration was treated as a fixed effect. The pen was treated as the experimental unit for the performance data, and the individual pig was treated as the experimental unit for data from tissue samples, statistical differences among mean values were assessed using Duncan’s multiple range test (*n* = 6). Data from cells were evaluated by one-way ANOVA followed by the Student-Newman-Keuls test. Figures were generated with GraphPad Prism 6, and 6 replicates were used per treatment for cell viability evaluation. For other data from cell experiments, 3 replicates were used. Effects were considered to be significant if *P* < 0.05.

## Results

### PQQ promotes growth performance and decreases the diarrhea incidence in weaned pigs

Pigs fed PQQ-supplemented diets exhibited a dose-related increase in ADG from d 0–14 (linear, *P* = 0.02) and across the entire experimental period (linear, *P* <  0.01), and their G:F improved across the entire experimental period (linear, *P* = 0.01). During d 0–28, compared to that in the CTRL group, the ADG of pigs fed diets supplemented with 0.15%, 0.30%, and 0.45% PQQ increased by 3.57%, 6.25% (*P* > 0.05) and 11.38% (*P* <  0.05), respectively. No difference in ADFI was observed among the dietary treatment groups (*P* > 0.05). The gain efficiency of pigs fed 0.15%, 0.30%, and 0.45% PQQ-supplemented diets were increased by 10.5%, 3.5% (*P* > 0.05) and 14.0% (*P* < 0.05), respectively, compared with that of pigs fed a diet without PQQ (Table 1). Compared to control pigs, pigs received the diet supplemented with 0.45% PQQ had a decreased diarrhea incidence during both d 0–14 and d 0–28 (*P* < 0.01). There was no significant difference in the diarrhea incidence among the treatment groups during d 15–28 (Table 2).

**Table 1:** Effects of dietary PQQ supplementation on growth performance in weaned pigs^1^

Items	Pyrroloquinoline quinone disodium levels,%				SEM	*P*-value		
	0	0.15	0.30	0.45		ANOVA	Linear	Quadratic
0 – 14 d								
ADG, g	345.31	349.41	372.10	384.29	12	0.09	0.02	0.83
ADFI, g	521.00	523.75	528.88	549.25	30	0.91	0.51	0.77
G:F	0.67	0.70	0.71	0.71	0.05	0.71	0.61	0.78
15 – 28 d								
ADG, g	555.00	568.00	568.75	594.63	32	0.83	0.43	0.85
ADFI, g	1074.25	1129.88	958.25	991.50	72	0.51	0.24	0.95
G:F	0.55	0.52	0.63	0.67	0.05	0.52	0.09	0.68
0 – 28 d								
ADG, g	447.54^a^	464.19^ab^	476.28^ab^	498.54^b^	12	0.02	< 0.01	0.82
ADFI, g	797.63	741.00	813.13	770.38	22	0.07	0.93	0.76
G:F	0.57^a^	0.63^ab^	0.59^ab^	0.65^b^	0.02	0.01	0.01	0.89

^1^ *SEM* standard error of the mean; the values are means ± SEM; 6 replicates. The linear and quadratic differences are with respect to pyrroloquinoline quinone disodium levels. Means within a row lacking a common letter are significantly different (*P* <  0.05)

**Table 2:** Effects of dietary PQQ supplementation on diarrhea incidence (%) in weaned pigs ^1^

Item	Pyrroloquinoline quinone disodium levels,%				SEM	*P*-value
	0	0.15	0.30	0.45		
0 – 14, d	23.07^a^	22.32^a^	18.60^a^	13.24^b^	1.71	< 0.01
15 – 28, d	8.04	6.70	4.91	4.91	1.23	0.08
0 – 28, d	15.55^a^	14.51^a^	11.76^ab^	9.08^b^	1.29	< 0.01

^1^ *SEM* standard error of the mean; the values are means ± SEM; 6 replicates. Means within a row lacking a common letter are significantly different (*P* < 0.05). The incidence of diarrhea in pigs within a pen was calculated as follows: ﻿Number of pigs with diarrhea in each pen × number of days of diarrhea/(total number of pigs × number of days in the experiment) × 100

### PQQ inhibits oxidative stress and improves gut morphology in weaned pigs

The levels of SOD in the liver, heart, and jejunum displayed positive improvements (linear, *P* < 0.01), and the concentration of MDA in the serum and jejunum was negatively related to the dose of PQQ (linear, *P* < 0.05). Compared with the basal diet, the diet supplemented with 0.15% PQQ increased the levels of GSH-Px in both the heart and jejunum and decreased the MDA content in the ileum (*P* < 0.05). Compared with control pigs, pigs received dietary supplementation with 0.3% PQQ exhibited increased SOD levels in the serum and jejunum; increased GSH-Px levels in the serum, liver, heart, jejunum and ileum; and decreased concentrations of MDA in the serum, liver and ileum (*P* < 0.05). Compared to pigs fed the basal diet, pigs that received the basal diet supplemented with 0.45% PQQ exhibited increased SOD activity in the liver and jejunum, increased GSH-Px levels in the serum and heart, and reduced MDA concentrations in the serum, liver, jejunum and ileum (*P* < 0.05, Tables 3 and 4).

**Table 3:** Effects of dietary PQQ supplementation on antioxidant activities in weaned pigs^1^

Items	Pyrroloquinoline quinone disodium levels,%				SEM	*P*-value		
	0	0.15	0.30	0.45		ANOVA	Linear	Quadratic
Serum								
SOD, U/mg	46.42^b^	47.04^b^	52.95^a^	50.24^ab^	1.37	0.01	0.09	0.23
GSH-Px, U/mg	727.62^b^	688.16^b^	812.73^a^	908.16^a^	35.90	0.02	0.06	0.19
MDA, nmol/mg	3.09^a^	2.52^ab^	2.30^b^	2.25^b^	0.20	< 0.01	< 0.01	0.07
CAT, IU/mg	3.14	4.00	4.57	3.62	0.48	0.21	0.36	0.07
Liver								
SOD, U/mg	156.16^b^	161.53^b^	183.95^ab^	222.86^a^	11.10	< 0.01	< 0.01	0.15
GSH-Px, U/mg	968.42^b^	974.70^b^	1041.32^a^	1029.44^ab^	17.14	< 0.01	0.12	0.49
MDA, nmol/mg	3.38^a^	3.12^a^	1.45^b^	1.64^b^	0.32	0.01	0.05	0.60
CAT, U/mg	38.12	39.88	40.71	45.39	4.71	0.73	0.30	0.76
Heart								
SOD, U/mg	163.34	171.12	184.78	193.39	8.40	0.09	0.01	0.96
GSH-Px, U/mg	500.78^b^	548.85^a^	544.61^a^	537.97^a^	19.24	< 0.01	0.09	0.06
MDA, nmol/mg	3.53	4.41	2.82	1.66	0.35	0.31	0.23	0.17
CAT, U/mg	4.16	3.92	4.35	4.31	1.04	0.99	0.85	0.92

^1^ *SEM* standard error of the mean; the values are means ± SEM; 6 replicates. The linear and quadratic differences are with respect to pyrroloquinoline quinone disodium levels. Means within a row lacking a common letter are significantly different (*P* < 0.05). Antioxidant activities are expressed per mg of protein

**Table 4:** Effects of dietary PQQ supplementation on antioxidant activities in the intestinal tissues of weaned pigs^1^

Items	Pyrroloquinoline quinone disodium levels,%				SEM	*P*-value		
	0	0.15	0.3	0.45		ANOVA	Linear	Quadratic
Duodenum								
SOD, U/mg	95.74	92.16	111.52	98.04	5.98	0.15	0.34	0.42
GSH-Px, U/mg	85.20	100.79	105.69	103.41	5.09	0.05	0.51	0.10
MDA, nmol/mg	0.67	0.90	0.68	0.68	0.06	0.05	0.08	0.09
CAT, U/mg	2.14	1.83	2.10	2.13	0.36	0.92	0.89	0.65
Jejunum								
SOD, U/mg	95.53^b^	106.08^ab^	114.80^a^	116.16^a^	4.60	0.02	< 0.01	0.33
GSH-Px, U/mg	102.32^b^	114.87^a^	119.05^a^	109.62^ab^	2.75	< 0.01	0.10	0.57
MDA, nmol/mg	0.79^a^	0.72^ab^	0.45^b^	0.40^b^	0.09	0.01	< 0.01	0.87
CAT, U/mg	2.22	1.86	2.54	2.43	0.39	0.64	0.46	0.75
Ileum								
SOD, U/mg	87.92	86.71	85.39	89.29	3.42	0.87	0.86	0.46
GSH-Px, U/mg	103.08	98.32	113.41	108.90	5.50	0.27	0.34	0.98
MDA, nmol/mg	1.35^a^	0.93^b^	0.81^b^	0.86^b^	0.11	0.01	0.20	0.05
CAT, U/mg	1.16	1.26	1.29	1.25	0.03	0.08	0.08	0.05

^1^ *SEM* standard error of the mean; the values are means ± SEM; 6 replicates. The linear and quadratic differences are with respect to pyrroloquinoline quinone disodium levels. Means within a row lacking a common letter are significantly different (*P* < 0.05). Antioxidant activities are expressed per mg of protein

Notably, the CD and VCR increased (quadratic, *P* < 0.01) in the jejunum as the percentage of supplementary PQQ increased. Compared with control pigs, pigs received the diet supplemented with 0.3% PQQ exhibited an increased VH in the duodenum and ileum, an increased VCR in the jejunum and ileum, and a decreased CD in the jejunum and ileum. Compared with control pigs, pigs received dietary supplementation with 0.45% PQQ exhibited a decreased CD in the ileum and an increased VCR in the jejunum (*P* < 0.05, Table 5).

**Table 5:** Effects of dietary PQQ supplementation on intestinal morphology in weaned pigs^1^

Item	Pyrroloquinoline quinone disodium levels,%				SEM	*P*-value		
	0	0.15	0.30	0.45		ANOVA	Linear	Quadratic
Duodenum								
Villus height, μm	332.00^b^	347.67^ab^	426.67^b^	366.50^ab^	22.30	0.04	0.09	0.11
Crypt depth, μm	281.17	268.00	209.83	213.83	26.50	0.17	0.05	0.75
Villus height/Crypt depth	1.22	1.36	2.22	1.99	0.29	0.08	0.06	0.53
Jejunum								
Villus height, μm	311.95	271.55	326.70	335.26	16.00	0.05	0.10	0.14
Crypt depth, μm	233.26^a^	181.51^ab^	135.27^b^	197.43^a^	15.00	< 0.01	0.11	< 0.01
Villus height/Crypt depth	1.40^b^	1.54^b^	2.46^a^	1.71^ab^	0.16	< 0.01	0.32	0.01
Ileum								
Villus height, μm	275.51^b^	256.47^b^	363.13^a^	289.20^ab^	19.00	< 0.01	0.10	0.17
Crypt depth, μm	197.39^a^	164.37^ab^	131.28^b^	138.07^b^	14.10	0.02	0.13	0.18
Villus height/Crypt depth	1.48^b^	1.58^b^	2.98^a^	2.56^a^	0.41	0.04	0.08	0.53

^1^ *SEM* standard error of the mean; the values are means ± SEM; 6 replicates. The linear and quadratic differences are with respect to pyrroloquinoline quinone disodium levels. Means within a row lacking a common letter are significantly different (*P* < 0.05)

### Optimal concentration and time point of H_2_O_2_ and PQQ treatment in IPEC-J2 cells

**Fig. 1:**
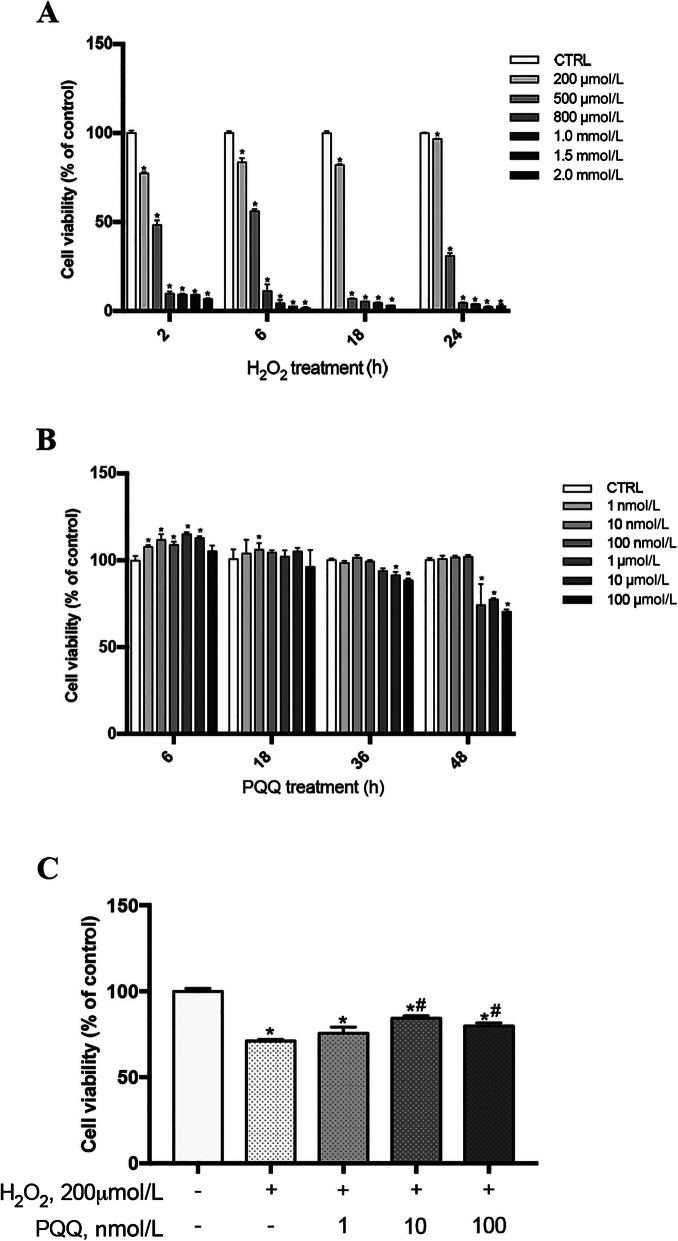
Effects of PQQ and H_2_O_2_ on cell viability *in vitro*. **a**, Cells were incubated with H_2_O_2_ for 2, 6, 18 and 24 h at concentrations of 0, 200 μmol/L, 500 μmol/L, 800 μmol/L, 1 mmol/L, 1.5 mmol/L and 2 mmol/L; **b**, Cells were incubated with PQQ at concentrations of 1 nmol/L, 10 nmol/L, 100 nmol/L, 1 μmol/L, 10 μmol/L and 100 μmol/L for 6, 18, 36 and 48 h; **c**, Cells were pretreated with PQQ for 6 h at concentrations of 1 nmol/L, 10 nmol/L, or 100 nmol/L and were then treated with both H_2_O_2_ (200 μmol/L) and PQQ for another 2 h. Cell viability in all treatment groups were evaluated with a CCK-8 assay. CTRL, cells cultured in basal medium; H_2_O_2_, cells cultured with 200 μmol/L H_2_O_2_; PQQ, cells cultured with 10 nmol/L PQQ for 6 h and then incubated with both 200 μmol/L H_2_O_2_ and 10 nmol/L PQQ for another 2 h. * denotes a significant difference (*P* < 0.05) with respect to the CTRL group. # denotes a significant difference (*P* < 0.05) with respect to the H_2_O_2_ group. *n =* 6

Incubation of cells with H_2_O_2_ for 2, 6, 18 and 24 h demonstrated that H_2_O_2_ significantly decreased cell viability at a concentration of 200 μmol/L and that the effect was dose-dependent at higher concentrations (*P* < 0.05, Fig. 1a). Therefore, IPEC-J2 cells treated with 200 μmol/L H_2_O_2_ for 2 h were used for the following experiments.

The viability of IPEC-J2 cells were determined after treatment with different concentrations of PQQ (0, 1 nmol/L, 10 nmol/L, 100 nmol/L, 1 μmol/L and 10 μmol/L) for 6, 18, 36 or 48 h. Low concentrations of PQQ (1 nmol/L, 10 nmol/L and 100 nmol/L) added to the cell culture medium did not cause cytotoxicity after 48 h of culture. Concentrations of 1 μmol/L had no negative effects on cell viability through 36 h but displayed direct toxic effects on cells (*P* < 0.05) at 48 h. However, high concentrations of PQQ (1 μmol/L and 10 μmol/L) significantly decreased cell viability beginning at 36 h of culture (Fig. 1b). Additionally, pretreatment with 10 nmol/L or 100 nmol/L PQQ for 6 h prior to incubation with PQQ and H_2_O_2_ for another 2 h reversed the negative effect of H_2_O_2_ on cell viability (*P* <  0.05, Fig. 1c). Based on these results, cells were pretreated with 10 nmol/L PQQ for 6 h prior to incubation with H_2_O_2_ (200 μmol/L) and PQQ (10 nmol/L) for another 2 h in further experiments.

### PQQ inhibits H_2_O_2_-induced oxidative cytokines and tight junction protein expression in IPEC-J2 cells

**Fig. 2:**
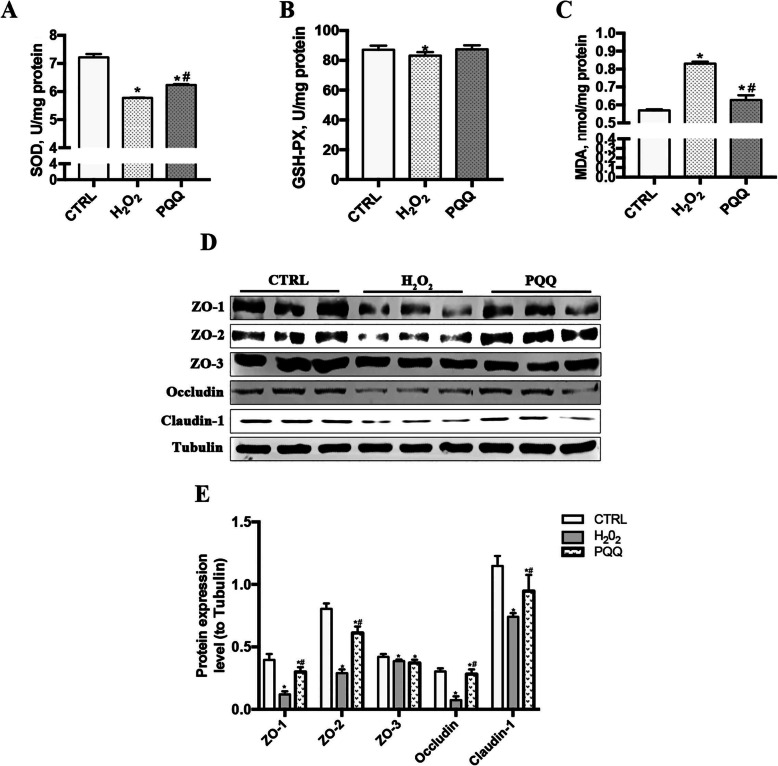
Effects of PQQ on oxidative cytokines and tight junction proteins in cells cultured with H_2_O_2_. **a-c**, Levels of SOD, GSH-Px and MDA in IPEC-J2 cells. **d**, Western blot analysis of ZO-1, ZO-2, ZO-3, occludin, and claudin-1. Densitometric values were normalized to those of Tubulin. **e**, Statistical analysis of the data in D. CTRL, cells cultured in basal medium; H_2_O_2_, cells cultured with 200 μmol/L H_2_O_2_; PQQ, cells cultured with 10 nmol/L PQQ for 6 h and then incubated with both 200 μmol/L H_2_O_2_ and 10 nmol/L PQQ for another 2 h. * denotes a significant difference (*P* < 0.05) with respect to the CTRL group. # denotes a significant difference (*P* < 0.05) with respect to the H_2_O_2_ group. *n =* 3

Compared to control treatment, treatment of IPEC-J2 cells with 200 μmol/L H_2_O_2_ at alone clearly decreased the levels of SOD and GSH-Px but increased the MDA concentration. In contrast, pretreatment of cells induced by H_2_O_2_ with 10 nmol/L PQQ resulted in increased SOD and GSH-Px levels and a decreased MDA concentration compared with those in the H_2_O_2_ group (*P* < 0.05, Fig. 2a-c).

Notably, H_2_O_2_ supplementation in the medium effectively decreased the protein expression levels of ZO-1, ZO-2, ZO-3, occludin, and claudin-1 in IPEC-J2 cells compared with CTRL group cells (*P* < 0.05). In contrast, cells pretreated with PQQ prior to treatment with both PQQ and H_2_O_2_ exhibited increased protein expression levels of ZO-1, ZO-2, Occludin and Claudin-1 but not ZO-3 (*P* > 0.05, Fig. 2d and e).

### PQQ decreases the ROS level and the expression levels of ROS-regulating proteins

**Fig. 3:**
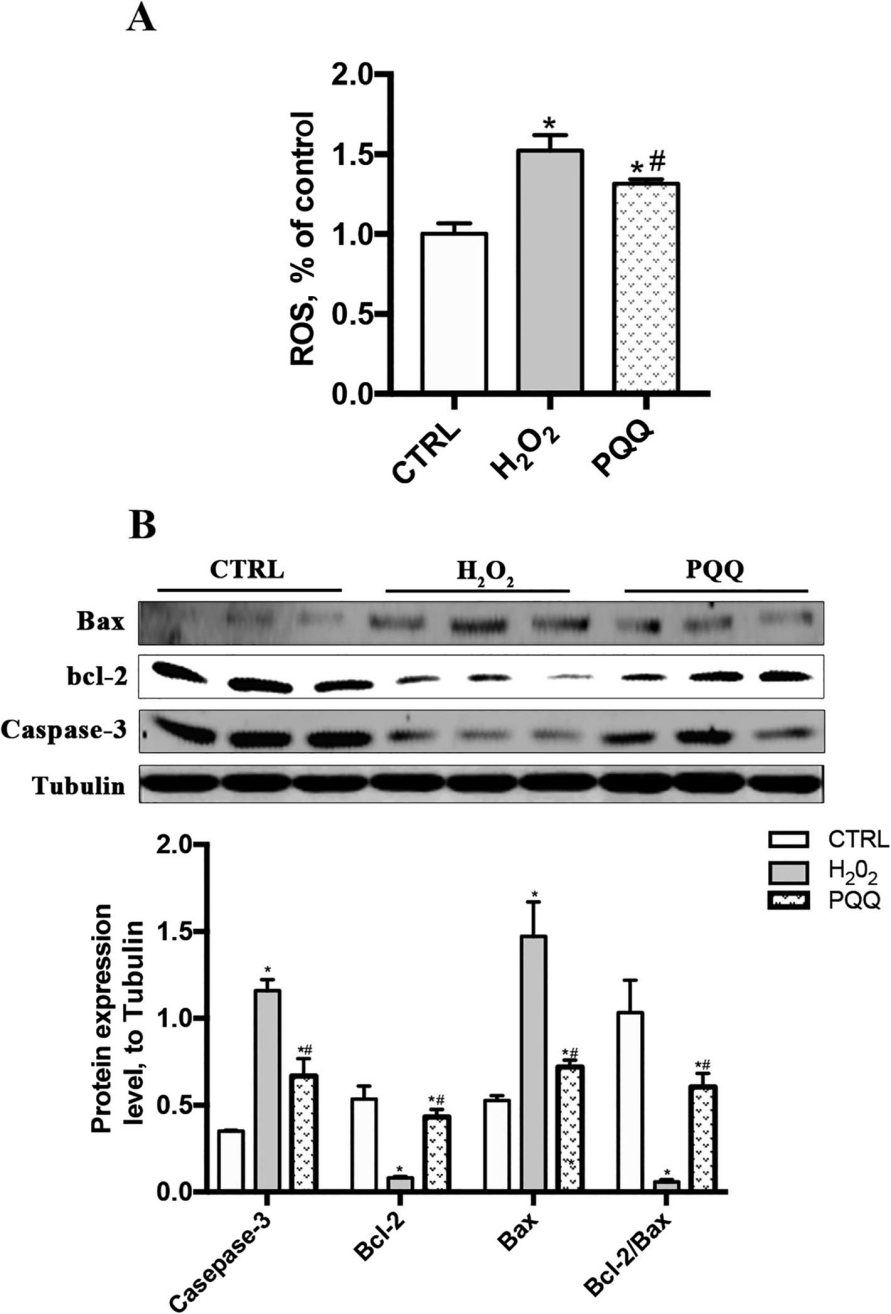
Effects of PQQ on levels of ROS and ROS-regulating proteins in cells cultured with H_2_O_2_. **a**, Level of ROS in IPEC-J2 cells. **b**, Western blot analysis of bax, bcl-2 and Caspase-3. Densitometric values were normalized to those of Tubulin. CTRL, cells cultured with basal medium; H_2_O_2_, cells cultured with 200 μmol/L H_2_O_2_ for 2 h; PQQ, cells cultured with 10 nmol/L PQQ for 6 h and then incubated with both 200 μmol/L H_2_O_2_ and 10 nmol/L PQQ for another 2 h. * denotes a significant difference (*P* < 0.05) with respect to the CTRL group. # denotes a significant difference (*P* < 0.05) with respect to the H_2_O_2_ group. *n* = 3

Compared to control treatment, H_2_O_2_ supplementation in the medium effectively increased the level of ROS and the protein expression levels of caspase-3 and bax but decreased the level of bcl-2 and the bcl-2/Bax ratio (*P* < 0.05). Pretreatment with PQQ in IPEC-J2 cells challenged with H_2_O_2_ resulted in a decrease in the ROS concentration and levels of Caspase-3 and Bax as well as an increase in the bcl-2 level and the bcl-2/Bax ratio compared with those in cells treated with H_2_O_2_ alone (*P* < 0.05, Fig. 3a and b).

### PQQ increases the activity of the Nrf2 signaling pathway

**Fig. 4:**
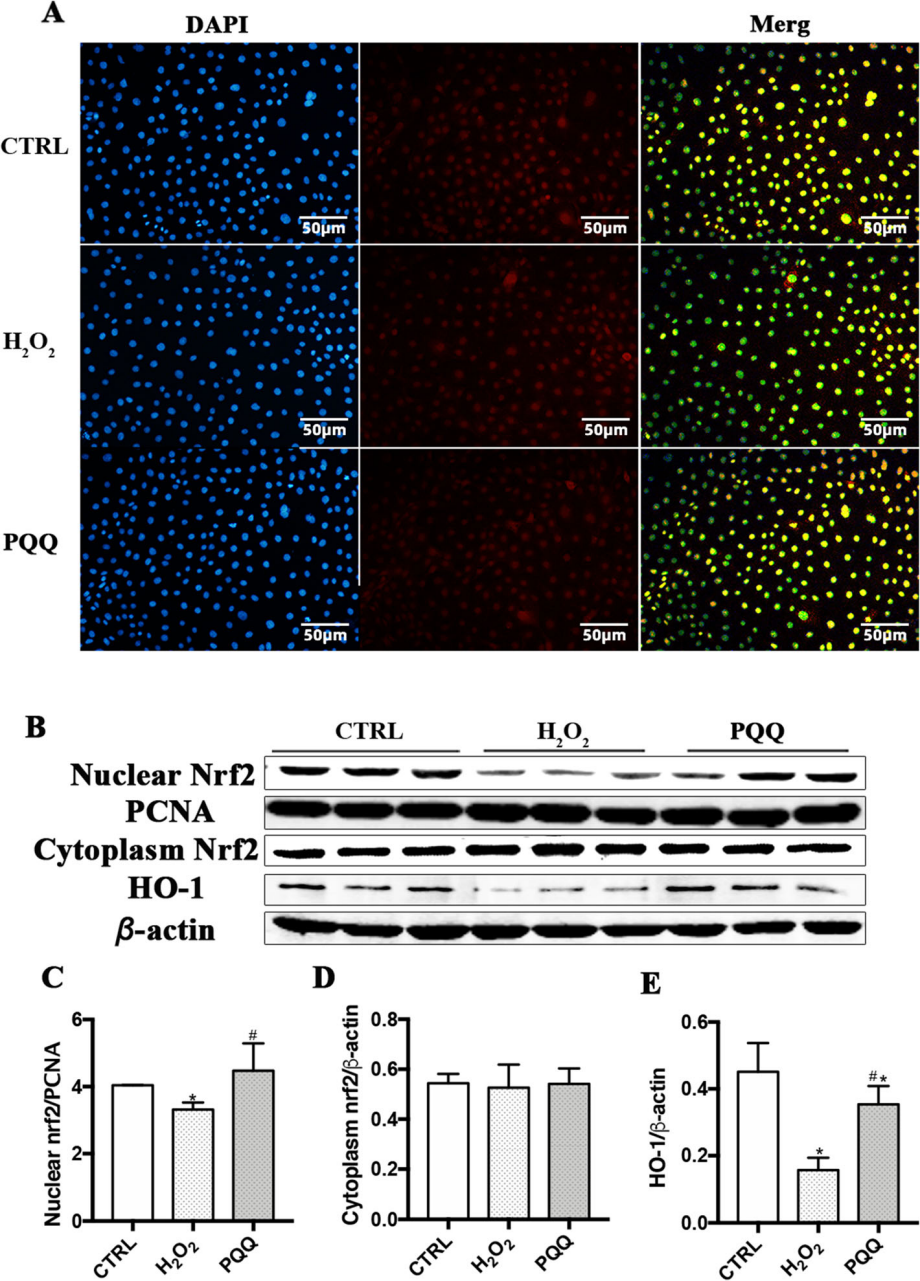
Effects of PQQ on proteins expression of Nrf2 and HO-1 in cells cultured with H_2_O_2_. **a**, Immunofluorescence staining of cytoplasmic and nuclear Nrf2 in IPEC-J2 cells. The scale bar represents 50 μm. **b**, Western blot analysis of nuclear Nrf2, cytoplasmic Nrf2, and HO-1. Densitometric values were normalized to those of PCNA or β-actin, as appropriate; **c-e**, Statistical analysis of the data in B. CTRL, cells cultured with basal medium; H_2_O_2_, cells cultured with 200 μmol/L H_2_O_2_ for 2 h; PQQ, cells cultured with 10 nmol/L PQQ for 6 h and then incubated with both 200 μmol/L H_2_O_2_ and 10 nmol/L PQQ for another 2 h. *n* = 3

To determine the mechanism by which PQQ decreases the sensitivity of IPEC-J2 cells to H_2_O_2_, the expression of Nrf2 and HO-1 were assessed. The fluorescence values of nuclear Nrf2 in cells incubated with H_2_O_2_ alone were significantly lower than those in CTRL cells. The fluorescence values of IPEC-J2 cells treated with both H_2_O_2_ and PQQ were significantly higher than those of cells incubated with H_2_O_2_ alone (Fig. 4a).

Additionally, compared with those in CTRL cells, the protein levels of nuclear Nrf2 and HO-1 in cells treated with H_2_O_2_ were decreased (*P* <  0.05). This decrease in the levels of nuclear Nrf2 and HO-1 (*P* < 0.05) were attenuated in the PQQ group compared with the H_2_O_2_ group (Fig. 4b-e).

### PQQ exhibits no protective effects on IPEC-J2 cells treated with Nrf2 siRNA

**Fig. 5:**
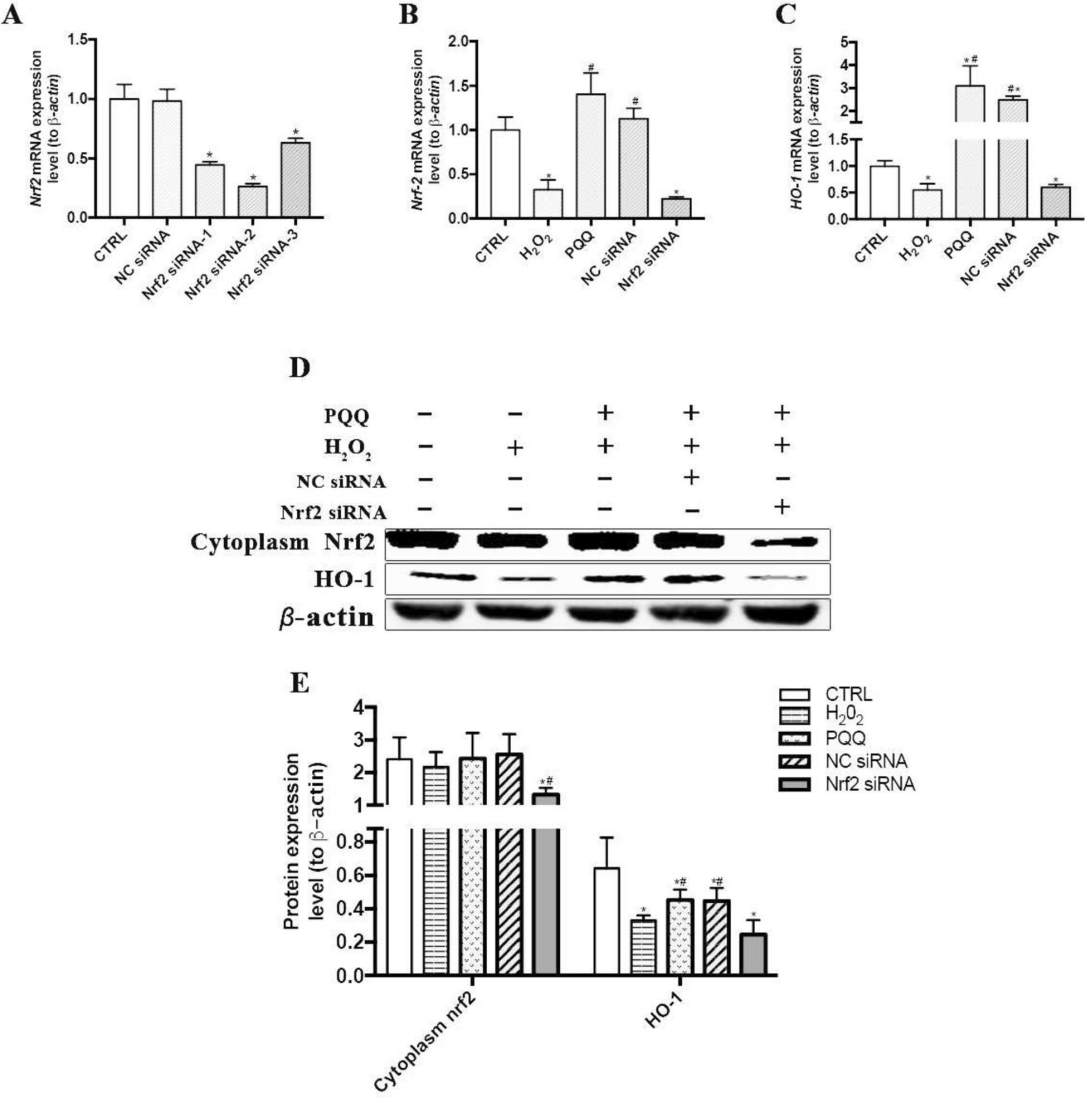
Effects of PQQ on levels of Nrf2 and HO-1 in cells treated with siRNA. **a** Nrf2 knockdown efficiency in cells transfected with three different sequences of Nrf2 siRNA or with NC siRNA. **b** and **c** mRNA expression levels of Nrf2 and HO-1 in IPEC-J2 cells transfected with or without Nrf2 siRNA-2. **d** Western blot analysis of Nrf2 and HO-1 in cells transfected with or without Nrf2 siRNA-2. **e** Statistical analysis of the data in D. CTRL, cells cultured with basal medium; H_2_O_2_, cells cultured with 200 μmol/L H_2_O_2_ for 2 h; PQQ, cells cultured with 10 nmol/L PQQ for 6 h and then incubated with both H_2_O_2_ (200 μmol/L) and PQQ (10 nmol/L) for another 2 h. NC siRNA, cells transfected with NC siRNA and then pretreated with PQQ for 6 h prior to incubation with both PQQ and H_2_O_2_ for another 2 h. Nrf2 siRNA, cells transfected with Nrf2 siRNA-2, pretreated with PQQ for 6 h, and then incubated with both PQQ and H_2_O_2_ for another 2 h. * denotes a significant difference (*P* < 0.05) with respect to the CTRL group. # denotes a significant difference (*P* < 0.05) with respect to the H_2_O_2_ group. *n =* 3

**Fig. 6:**
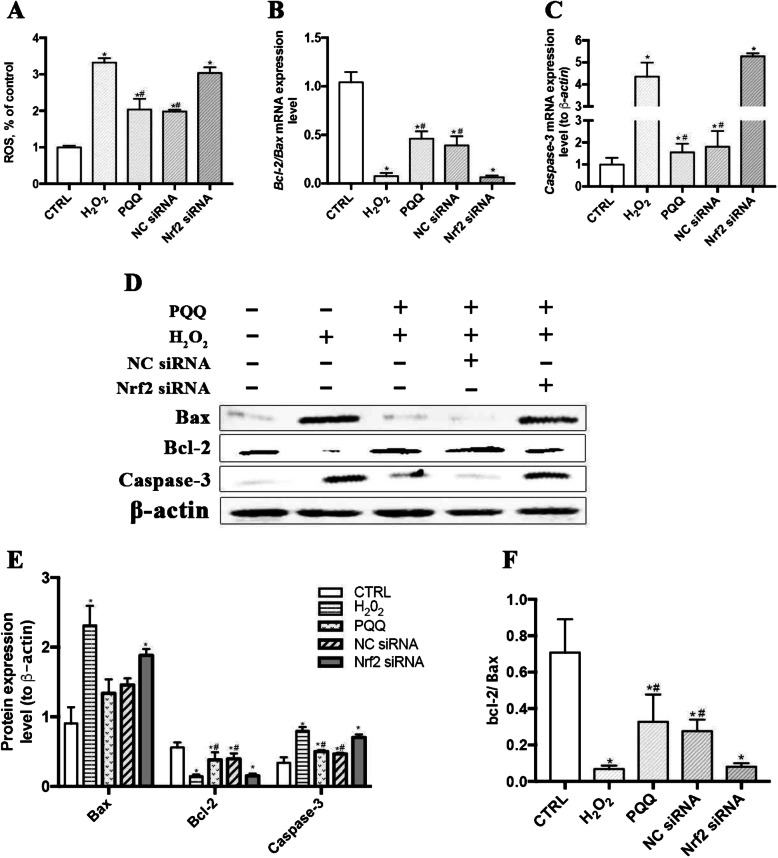
Effects of PQQ on levels of ROS and ROS-regulated proteins in cells treated with siRNA. **a** Level of ROS in IPEC-J2 cells treated with or without Nrf2 siRNA-2. **b-c** Ratio of Bcl-2/Bax mRNA levels and mRNA expression levels of Caspase-3 in IPEC-J2 cells treated with or without Nrf2 siRNA-2. **d** Western blot analysis of cytoplasmic Bax, Bcl-2 and Caspase-3. Densitometric values were normalized to those of β-actin. **e** Statistical analysis of the data in D. **f** Statistical analysis of the Bcl-2/Bax protein ratio. CTRL, cells cultured with basal medium; H_2_O_2_, cells cultured with 200 μmol/L H_2_O_2_ for 2 h; PQQ, cells cultured with 10 nmol/L PQQ for 6 h and then incubated with both H_2_O_2_ (200 μmol/L) and PQQ (10 nmol/L) for another 2 h. NC siRNA, cells transfected with NC siRNA and then pretreated with PQQ for 6 h prior to incubation with both PQQ and H_2_O_2_ for another 2 h. Nrf2 siRNA, cells transfected with Nrf2 siRNA-2, pretreated with PQQ for 6 h, and then incubated with both PQQ and H_2_O_2_ for another 2 h. *n* = 3

**Fig. 7:**
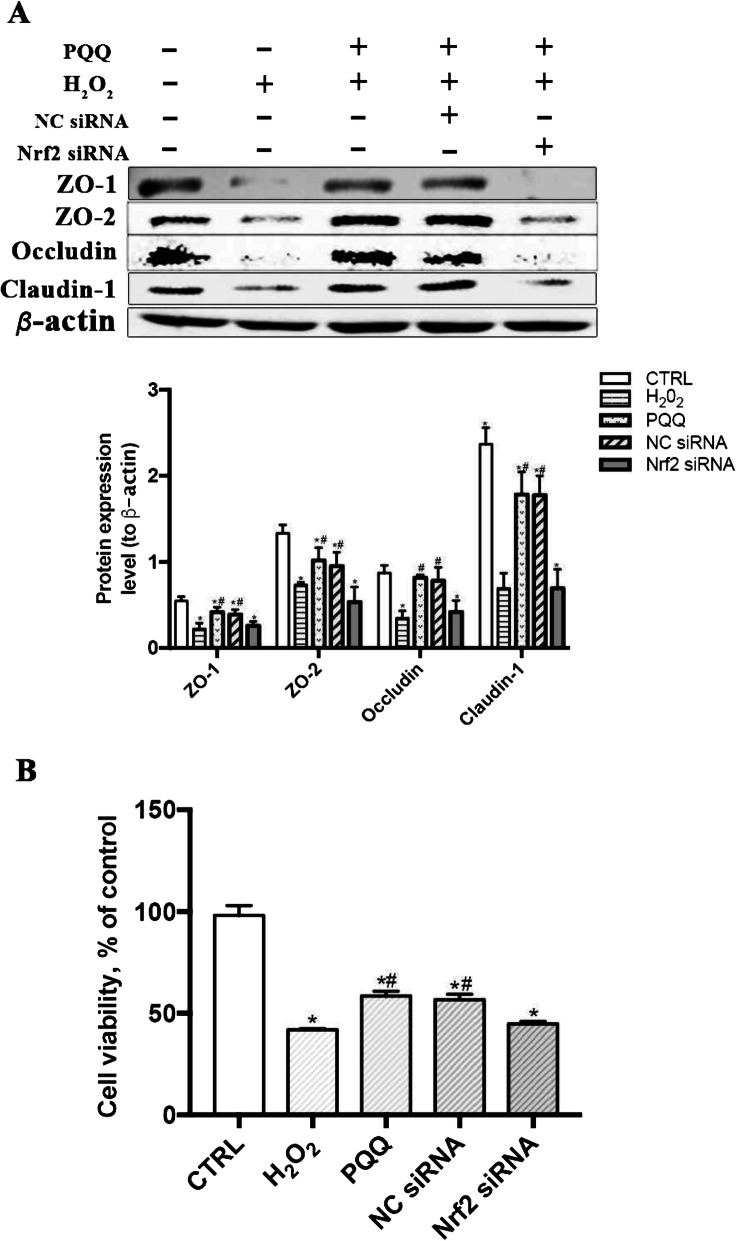
Effects of PQQ on tight junctions expression and cell viability in cells treated with siRNA. **a** Western blot analysis of ZO-1, ZO-2, Occludin and Claudin-1 in IPEC-J2 cells treated with or without Nrf2 siRNA-2. The densitometric values were normalized to those of β-actin. *n* = 3. **b** Viability of IPEC-J2 cells treated with or without Nrf2 siRNA-2 and incubated in the presence or absence of H_2_O_2_ or PQQ. CTRL, cells cultured with basal medium; H_2_O_2_, cells cultured with 200 μmol/L H_2_O_2_ for 2 h; PQQ, cells cultured with 10 nmol/L PQQ for 6 h and then incubated with both H_2_O_2_ (200 μmol/L) and PQQ (10 nmol/L) for another 2 h. NC siRNA, cells transfected with NC siRNA and then pretreated with PQQ for 6 h prior to incubation with both PQQ and H_2_O_2_ for another 2 h. Nrf2 siRNA, cells transfected with Nrf2 siRNA-2, pretreated with PQQ for 6 h, and then incubated with both PQQ and H_2_O_2_ for another 2 h. *n* = 6. * denotes a significant difference (*P* < 0.05) with respect to the CTRL group. # denotes a significant difference (*P* < 0.05) with respect to the H_2_O_2_ group

The mRNA and protein expression levels of Nrf2 were significantly reduced (*P* <  0.05) in the Nrf2 siRNA-1-, Nrf2 siRNA-2-, and Nrf2 siRNA-3-treated groups compared with the CTRL group. The highest Nrf2 knockdown efficiency in IPEC-J2 cells was achieved with Nrf2 siRNA-2; thus, we used Nrf2 siRNA-2 in subsequent experiments (Fig. 5a). Pretreatment of IPEC-J2 cells with Nrf2 siRNA largely abrogated the beneficial function of PQQ pretreatment, as indicated by the decreased mRNA and protein levels of Nrf2 and HO-1 (*P* < 0.05, Fig. 5b-d). Moreover, the reduction in the intracellular ROS level and changes in mRNA and protein expression levels of caspase-3, bax and bcl-2 induced by PQQ pretreatment in cells cultured with H_2_O_2_ were abolished after transfection with Nrf2 siRNA (*P* < 0.05, Fig. 6). Treatment with Nrf2 siRNA largely abrogated the beneficial effects of PQQ pretreatment on the H_2_O_2_-induced decreases in cell viability and the protein expression levels of ZO-1, ZO-2, Occludin and Claudin-1 (*P* < 0.05, Fig. 7); however, none of these effects were observed in the NC siRNA treatment group.

## Discussion

Oxidative stress often leads to structural injury to the gut and resulting diarrhea in pigs after weaning. PQQ supplementation in the diet improves growth performance in weaned mice [32] and birds [33] and promotes jejunal health in weaned pigs [34]. In our previous research, pigs fed diets supplemented with PQQ displayed improved growth performance, small intestinal morphology, and antioxidant capacity and decreased diarrhea incidence during the weaning transition [18]. In the present study, we confirmed that dietary PQQ can decrease oxidative injury induced by weaning and determined the mechanism through which PQQ supplementation improves antioxidant function in weaned pigs. In this study, the sample size of 6 replicates (*n* = 6) is proper for detecting the effect of PQQ on intestinal barrier function and immune response, however, as for growth performance, it warrants continual test with greater population of piglets.

The small intestinal epithelium is an absorptive barrier for nutrient transport and is the first defensive barrier against harmful substances [35]. Many studies have reported that H_2_O_2_ is a central redox signaling molecule that induces oxidative stress in intestinal epithelial cells, such as IPEC-J2 cells [36–38].

Tight junction proteins, including claudin proteins, zonula occludens proteins and occludin, are the main components providing barrier function in epithelial cells and maintain epithelial permeability and integrity [39]. Oxidative stress-induced alterations in tight junction protein levels can reduce the viability of porcine intestinal epithelial cells [40]. In the present study, we found that H_2_O_2_ exposure decreased the expression levels of ZO-1, ZO-2, occludin and claudin-1 in IPEC-J2 cells and decreased the viability of these cells, consistent with previous research [41].

Extracellular H_2_O_2_ stimulates high levels of intracellular ROS production and induces cell death caused by excess ROS [42, 43]. As MDA is the end product of lipid peroxidation, its concentration is closely tied to the extent of oxidative stress-induced cell damage via alteration of cell membrane permeability and uncoupling of oxidative phosphorylation in mitochondria [44]. Normally, excessive ROS in cells can cause oxidative stress to increase production of MDA, resulting in cytotoxicity. In addition, GSH-Px and SOD are considered the main enzymes in the antioxidant system that scavenge ROS [45]. In the present study, we demonstrated that H_2_O_2_ exposure caused the levels of SOD and GSH-Px to decrease and the levels of MDA and intracellular ROS to increase in IPEC-J2 cells, consistent with previous studies [46, 47].

Excessive production of ROS combined with an increase in the concentration of MDA can initiate programmed cell death [48]. A decreased Bcl-2/Bax ratio and the release of activated caspase-3 from mitochondrial cyt-c to the cytoplasm are markers of the programmed cell death process [49, 50]. Consistent with this result, our data demonstrated that H_2_O_2_-treated IPEC-J2 cells had a lower Bcl-2/Bax ratio and higher levels of total caspase-3 than CTRL cells. These results indicated that we successfully established a model of oxidative damage in IPEC-J2 cells.

Low supplementary concentrations of PQQ in cell media did not have a negative effect on cell viability [51, 52]. In the present study, we showed that PQQ concentrations of less than 100 nmol/L had no negative effects on cell viability at 48 h. Pretreatment with 10 nmol/L PQQ resulted in higher cell viability than pretreatment with the other concentrations of PQQ in IPEC-J2 cells cultured with H_2_O_2_. Additionally, the examined parameters (ZO-1, ZO-2, occludin, claudin-1, ROS, MDA, GSH-Px and SOD) were effectively restored by pretreatment with 10 nmol/L PQQ prior to exposure to H_2_O_2_. Therefore, we confirmed that supplementation at a suitable concentration attenuates intestinal injury by regulating intestinal redox reactions.

Protection of gut tight junction proteins from damage and intestinal epithelial cells from unnatural death induced by oxidative stress are often associated with the activation of antioxidant signaling pathways. The Nrf2 pathway is a master regulator of cell resistance to oxidative injury [53] Keap1 is a cytoplasmic junction protein that may downregulate Nrf2. Normally, under physiological conditions, of low the activity of Nrf2 activity is maintained at a low level via its distribution in the cytoplasm in the Nrf2-keap1 complex [54]. After activation, Nrf2 is phosphorylated, dissociates from the complex, and is then released to translocate into the nucleus, where it induces downstream expression of HO-1 and SOD, which eliminate ROS [55]. To further identify the pathway through which PQQ supplementation protects cells from oxidative stress, we determined the key genes involved in the Nrf2-mediated antioxidant response. Our results showed that PQQ supplementation effectively restored the stimulated oxidative status and increased the levels of nuclear Nrf2 and HO-1. Additionally, all of these positive effects of PQQ on cytotoxicity and oxidative stress damage induced by H_2_O_2_ were abolished due to siRNA-mediated knockdown of Nrf-2 in IPEC-J2 cells. Thus, we demonstrated that Nrf2/HO-1 are involved in the cytoprotective effects of PQQ on H_2_O_2_-induced damage in IPEC-J2 cells.

## Conclusion

In conclusion, we demonstrated that PQQ administration attenuates oxidative stress in weaned pigs, as evidenced by increased levels of antioxidant enzymes and tight junction proteins and decreased levels of ROS and MDA in tissues and cells. This positive effect of PQQ is associated with activation of the Nrf2/HO-1 pathway.

## Supplementary Information

**Additional file 1: Supplemental Table 1.** Ingredient composition of experimental diets (as-fed basis). **Supplemental Table 2.** Nutrient concentration of experimental diets (as-fed basis). **Supplemental Table 3.** Information of primary antibodies used for western blotting analysis. **Supplemental Table 4**. Primer sequences of target and reference genes.

## Data Availability

The data analyzed during the current study are available from the corresponding author on reasonable request.

## Abbreviations

PQQ: Pyrroloquinoline quinone
IPEC-J2: Porcine intestinal epithelial cell line
HO-1: Heme oxygenase-1
ADG: Average daily gain
ADFI: Average daily feed intake
G:F: Gain-to-feed ratio
VH: Villus height
CD: Crypt depth
VCR: Villus height/crypt depth ratio
GSH-Px: Glutathione peroxidase
SOD: Superoxide dismutase
MDA: Malondialdehyde
CAT: Catalase
siRNA: Small interfering RNA
ZO-1: Zonula occludens protein 1
ZO-2: Zonula occludens protein 2
ZO-3: Zonula occludens-3

## References

[1] Cappelli APG, Zoppi CC, Silveira LR, Batista TM, Paula FM, da Silva PMR, Rafacho A, Barbosa-Sampaio HC, Boschero AC, Carneiro EM (2018). Reduced glucose-induced insulin secretion in low-protein-fed rats is associated with altered pancreatic islets redox status. J Cell Physiol.

[2] Ranade R, Talukder S, Muscatello G, Celi P (2014). Assessment of oxidative stress biomarkers in exhaled breath condensate and blood of dairy heifer calves from birth to weaning. Vet J.

[3] Zhu LH, Zhao KL, Chen XL, Xu JX (2012). Impact of weaning and an antioxidant blend on intestinal barrier function and antioxidant status in pigs. J Anim Sci.

[4] Negroni A, Cucchiara S, Stronati L (2015). Apoptosis, necrosis, and necroptosis in the gut and intestinal homeostasis. Mediat Inflamm.

[5] Zhang C, Wang N, Xu Y, Tan HY, Li S, Feng Y (2018). Molecular mechanisms involved in oxidative stress-associated liver injury induced by chinese herbal medicine: an experimental evidence-based literature review and network pharmacology study. Int J Mol Sci.

[6] Hampson DJ (1986). Alterations in piglet small intestinal structure at weaning. Res Vet Sci.

[7] Hedemann MS, Højsgaard S, Jensen BB (2003). Small intestinal morphology and activity of intestinal peptidases in piglets around weaning. J Anim Physiol Anim Nutr (Berl).

[8] Erik K, Knudsen B (2001). Development of antibiotic resistance and options to replace antimicrobials in animal diets. Proc Nutr Soc.

[9] Stites TE, Mitchell AE, Rucker RB (2000). Physiological importance of quinoenzymes and the O-quinone family of cofactors. J Nutr.

[10] Akagawa M, Nakano M, Ikemoto K (2016). Recent progress in studies on the health benefits of pyrroloquinoline quinone. Biosci Biotechnol Biochem.

[11] Ohwada K, Takeda H, Yamazaki M, Isogai H, Nakano M, Shimomura M, Fukui K, Urano S (2008). Pyrroloquinoline Quinone (PQQ) prevents cognitive deficit caused by oxidative stress in rats. J Clin Biochem Nutr.

[12] Misra HS, Rajpurohit YS, Khairnar NP (2012). Pyrroloquinoline-quinone and its versatile roles in biological processes. J Biosci.

[13] Tao R, Karliner JS, Simonis U, Zheng J, Zhang J, Honbo N, Alano CC (2007). Pyrroloquinoline quinone preserves mitochondrial function and prevents oxidative injury in adult rat cardiac myocytes. Biochem Biophys Res Commun.

[14] Xu F, Yu H, Liu J, Cheng L (2014). Pyrroloquinoline quinone inhibits oxygen/glucose deprivation-induced apoptosis by activating the PI3K/AKT pathway in cardiomyocytes. Mol Cell Biochem.

[15] Wang J, Zhang HJ, Xu L, Long C, Samuel KG, Yue HY, Sun LL, Wu SG, Qi GH (2016). Dietary supplementation of pyrroloquinoline quinone disodium protects against oxidative stress and liver damage in laying hens fed an oxidized sunflower oil-added diet. Animal..

[16] Friedman JE, Dobrinskikh E, Alfonso-Garcia A, Fast A, Janssen RC, Soderborg TK, Anderson AL, Reisz JA, D'Alessandro A, Frank DN, Robertson CE, de la Houssaye BA, Johnson LK, Orlicky DJ, Wang XX, Levi M, Potma EO, el Kasmi KC, Jonscher KR (2018). Pyrroloquinoline quinone prevents developmental programming of microbial dysbiosis and macrophage polarization to attenuate liver fibrosis in offspring of obese mice. Hepatol Commun.

[17] Zhang B, Wang C, Yang W, Zhang H, Meng Q, Shi B, Shan A (2019). Transcriptome analysis of the effect of pyrroloquinoline quinone disodium (PQQ) on reproductive performance in sows during gestation and lactation. J Anim Sci Biotechnol.

[18] Yin X, Ming D, Bai L, Wu F, Liu H, Chen Y, Sun L, Wan Y, Thacker PA, Wu G, Wang F (2019). Effects of pyrroloquinoline quinone supplementation on growth performance and small intestine characteristics in weaned pigs. J Anim Sci.

[19] Zhang J, Wang X, Vikash V, Ye Q, Wu D, Liu Y, Dong W (2016). ROS and ROS-mediated cellular signaling. Oxidative Med Cell Longev.

[20] Deshmukh P, Unni S, Krishnappa G, Padmanabhan B (2017). The Keap1-Nrf2 pathway: promising therapeutic target to counteract ROS-mediated damage in cancers and neurodegenerative diseases. Biophys Rev.

[21] Zhao Y, Niu Y, He J, Zhang L, Wang C, Wang T (2019). Dietary dihydroartemisinin supplementation attenuates hepatic oxidative damage of weaned piglets with intrauterine growth retardation through the Nrf2/ARE signaling pathway. Animals (Basel).

[22] Wang Z, Han N, Zhao K, Li Y, Chi Y, Wang B (2019). Protective effects of pyrroloquinoline quinine against oxidative stress-induced cellular senescence and inflammation in human renal tubular epithelial cells via Keap1/Nrf2 signaling pathway. Int Immunopharmacol.

[23] National Research Council (2012). Guide for the care and use of laboratory animals.

[24] Marquardt RR, Jin LZ, Kim JW, Fang L, Frohlich AA, Baidoo SK (1999). Passive protective effect of egg-yolk antibodies against enterotoxigenic Escherichia coli K88+ infection in neonatal and early-weaned piglets. FEMS Immunol Med Microbiol.

[25] Ou D, Li D, Cao Y, Li X, Yin J, Qiao S, Wu G (2007). Dietary supplementation with zinc oxide decreases expression of the stem cell factor in the small intestine of weanling pigs. J Nutr Biochem.

[26] Huang C, Wang Y, He X, Jiao N, Zhang X, Qiu K, Piao X, Yin J (2019). The involvement of NF-κB/P38 pathways in scutellaria baicalensisextracts attenuating of Escherichia coli K88-induced acute intestinal injury in weaned piglets. Br J Nutr.

[27] Liu B, Jiang X, Cai L, Zhao X, Dai Z, Wu G, Li X (2019). Putrescine mitigates intestinal atrophy through suppressing inflammatory response in weanling piglets. J Anim Sci Biotechnol.

[28] Huang C, Ming D, Wang W, Wang Z, Hu Y, Ma X, Wang F (2020). Pyrroloquinoline quinone alleviates jejunal mucosal barrier function damage and regulates colonic microbiota in piglets challenged with enterotoxigenic Escherichia coli. Front Microbiol.

[29] Xiao K, Liu C, Tu Z (2020). Activation of the NF-κB and MAPK signaling pathways contributes to the inflammatory responses, but not cell injury, in IPEC-1 cells challenged with hydrogen peroxide. Oxidative Med Cell Longev.

[30] Pfaffl MW, Horgan GW, Dempfle L. Relative expression software tool (REST) for group-wise comparison and statistical analysis of relative expression results in real-time PCR. Nucleic Acids Res 2002: 30(9): e36. doi: 10.1093/nar/30.9.e36, 36e, 336. 10.1093/nar/30.9.e36 PMC113859 11972351

[31] Zhang X, Wang L, Qiu K, Xu D, Yin J (2019). Dynamic membrane proteome of adipogenic and myogenic precursors in skeletal muscle highlights EPHA2 may promote myogenic differentiation through ERK signaling. FASEB J.

[32] Steinberg F, Stites TE, Anderson P, Storms D, Chan I, Eghbali S, Rucker R (2003). Pyrroloquinoline quinone improves growth and reproductive performance in mice fed chemically defined diets. Exp Biol Med (Maywood).

[33] Wang J, Zhang HJ, Samuel KG, Long C, Wu SG, Yue HY, Sun LL, Qi GH (2015). Effects of dietary pyrroloquinoline quinone disodium on growth, carcass characteristics, redox status, and mitochondria metabolism in broilers. Poult Sci.

[34] Zhang H, Li J, Cao C, Zhang B, Yang W, Shi B, Shan A (2020). Pyrroloquinoline quinone inhibits the production of inflammatory cytokines via the SIRT1/NF-κB signal pathway in weaned piglet jejunum. Food Funct.

[35] Groschwitz KR, Hogan SP (2009). Intestinal barrier function: molecular regulation and disease pathogenesis. J Allergy Clin Immunol.

[36] Cao S, Wang C, Yan J, Li X, Wen J, Hu C (2020). Curcumin ameliorates oxidative stress-induced intestinal barrier injury and mitochondrial damage by promoting Parkin dependent mitophagy through AMPK-TFEB signal pathway. Free Radic Biol Med.

[37] Xu C, Qiao L, Ma L, Yan S, Guo Y, Dou X, Zhang B, Roman A (2019). Biosynthesis of polysaccharides-capped selenium nanoparticles using Lactococcus lactisNZ9000 and their antioxidant and anti-inflammatory activities. Front Microbiol.

[38] Chen Z, Yuan Q, Xu G, Chen H, Lei H, Su J (2018). Effects of quercetin on proliferation and H_2_O_2_-induced apoptosis of intestinal porcine enterocyte cells. Molecules..

[39] Lee B, Moon KM, Kim CY (2018). Tight junction in the intestinal epithelium: its association with diseases and regulation by phytochemicals. J Immunol Res.

[40] Luo R, Yang Q, Huang X, Yan Z, Gao X, Wang W, Xie K, Wang P, Gun S (2020). Clostridium perfringens beta2 toxin induced *in vitro* oxidative damage and its toxic assessment in porcine small intestinal epithelial cell lines. Gene..

[41] Zhuang Y, Wu H, Wang X, He J, He S, Yin Y (2019). Resveratrol attenuates oxidative stress-induced intestinal barrier injury through PI3K/Akt-mediated Nrf2 signaling pathway. Oxidative Med Cell Longev.

[42] Dong Y, Hou Q, Lei J, Wolf PG, Ayansola H, Zhang B (2020). Quercetin alleviates intestinal oxidative damage induced by H_2_O_2_ via modulation of GSH: *In vitro* screening and *in vivo* evaluation in a colitis model of mice. ACS Omega.

[43] Tanida S, Mizoshita T, Mizushima T, Sasaki M, Shimura T, Kamiya T, Kataoka H, Joh T (2011). Involvement of oxidative stress and mucosal addressin cell adhesion molecule-1 (MAdCAM-1) in inflammatory bowel disease. J Clin Biochem Nutr.

[44] Fentoğlu Ö, Kırzıoğlu FY, Bulut MT, Kumbul Doğuç D, Kulaç E, Önder C, Günhan M (2015). Evaluation of lipid peroxidation and oxidative DNA damage in patients with periodontitis and hyperlipidemia. J Periodontol.

[45] Zou Y, Wang J, Peng J, Wei H (2016). Oregano essential oil induces SOD1 and GSH expression through Nrf2 activation and alleviates hydrogen peroxide-induced oxidative damage in IPEC-J2 cells. Oxidative Med Cell Longev.

[46] Xiao H, Wu M, Shao F, Guan G, Huang B, Tan B, Yin Y (2016). N-acetyl-L-cysteine protects the enterocyte against oxidative damage by modulation of mitochondrial function. Mediat Inflamm.

[47] Yuan Z, Liang Z, Yi J, Chen X, Li R, Wu Y, Wu J, Sun Z (2019). Protective effect of koumine, an alkaloid from gelsemium sempervirens, on injury induced by H_2_O_2_ in IPEC-J2 cells. Int J Mol Sci.

[48] Green DR (2000). Apoptotic pathways: paper wraps stone blunts scissors. Cell.

[49] Youle RJ, Strasser A (2008). The BCL-2 protein family: opposing activities that mediate cell death. Nat Rev Mol Cell Biol.

[50] Liu Z, Sun C, Tao R, Xu X, Xu L, Cheng H, Wang Y, Zhang D (2016). Pyrroloquinoline quinone decelerates rheumatoid arthritis progression by inhibiting inflammatory responses and joint destruction via modulating NF-κB and MAPK pathways. Inflammation..

[51] Guan S, Xu J, Guo Y, Ge D, Liu T, Ma X, Cui Z (2015). Pyrroloquinoline quinone against glutamate-induced neurotoxicity in cultured neural stem and progenitor cells. Int J Dev Neurosci.

[52] Nakano M, Suzuki H, Imamura T, Lau A, Lynch B (2013). Genotoxicity of pyrroloquinoline quinone (PQQ) disodium salt (BioPQQ™). Regul Toxicol Pharmacol.

[53] Wen Z, Liu W, Li X, Chen W, Liu Z, Wen J, Liu Z (2019). A protective role of the NRF2-Keap1 pathway in maintaining intestinal barrier function. Oxidative Med Cell Longev.

[54] Katsuoka F, Otsuki A, Takahashi M, Ito S, Yamamoto M (2019). Direct and specific functional evaluation of the Nrf2 and mafG heterodimer by introducing a tethered dimer into small Maf-deficient cells. Mol Cell Biol.

[55] Li B, Nasser MI, Masood M, Adlat S, Huang Y, Yang B, Luo C, Jiang N (2020). Efficiency of traditional chinese medicine targeting the Nrf2/HO-1 signaling pathway. Biomed Pharmacother.

