# Supplementary material for: Pyrroloquinoline quinone regulates the redox status in vitro and in vivo of weaned pigs via the Nrf2/HO-1 pathway
Source: J Anim Sci Biotechnol. 2021 Jun 18;12:77. doi: 10.1186/s40104-021-00595-x (PMC8212497; doi:10.1186/s40104-021-00595-x)
Supplement: Supplementary file 1 — Additional file 1: Supplemental Table 1. Ingredient composition of experimental diets (as-fed basis). Supplemental Table 2. Nutrient concentration of experimental diets (as-fed basis). Supplemental Table 3. Information of primary antibodies used for western blotting analysis. Supplemental Table 4. Primer sequences of target and reference genes. [file 40104_2021_595_MOESM1_ESM.doc]

**Supplementary data**

**Supplemental Table 1. Ingredient composition of experimental diets (as-fed basis)**

| Items | Pyrroloquinoline quinone disodium levels (%) | | | |
| --- | --- | --- | --- | --- |
|  | 0 | 0.15 | 0.30 | 0.45 |
| Ingredients (%) |  |  |  |  |
| Corn | 60.05 | 59.90 | 59.75 | 59.60 |
| Soybean meal (43% CP) | 14.00 | 14.00 | 14.00 | 14.00 |
| Extruded soybean | 14.08 | 14.08 | 14.08 | 14.08 |
| Fish meal (64% CP) | 3.00 | 3.00 | 3.00 | 3.00 |
| Dried whey | 4.00 | 4.00 | 4.00 | 4.00 |
| Limestone | 0.35 | 0.35 | 0.35 | 0.35 |
| Dicalcium phosphate | 1.80 | 1.80 | 1.80 | 1.80 |
| Salt | 0.34 | 0.34 | 0.34 | 0.34 |
| L-Lysine·HCL (78.8%) | 0.50 | 0.50 | 0.50 | 0.50 |
| L-Threonine (98.5%) | 0.18 | 0.18 | 0.18 | 0.18 |
| L-Tryptophan (98.5%) | 0.05 | 0.05 | 0.05 | 0.05 |
| L-Methionine (99.0%) | 0.15 | 0.15 | 0.15 | 0.15 |
| Glucose | 0.50 | 0.50 | 0.50 | 0.50 |
| Vitamin and mineral premix^1^ | 1.00 | 1.00 | 1.00 | 1.00 |
| Pyrroloquinoline quinone disodium^2^ | 0.00 | 0.15 | 0.30 | 0.45 |

^1^Premix provided the following per kg: vitamin A, 1,000,000 IU; vitamin D_3_, 230,000 IU; vitamin E, 2,000 IU; vitamin K_3_, 240 mg; thiamin, 150 mg; riboflavin, 400 mg; pyridoxine, 300 mg; vitamin B_12_, 1,200 μg; niacin, 3,000 mg; pantothenic acid, 1,300 mg; folic acid, 75 mg; biotin, 5 mg; choline chlorine, 40 mg; Fe, 9 g; Cu, 10mg; Zn, 90 mg; Mn, 2 mg; I, 32 mg; Se, 0.3 mg.

^2^PQQ·Na_2_ (purity, ≥ 98%) was provided by Shanghai Medical Life Sciences Research Center Co. Ltd (Shanghai, China), and was diluted with corn starch to a concentration of 1 g/kg mixture before being mixed into the diet.

**Supplemental Table 2. Nutrient concentration of experimental diets (as-fed basis)**

| Item | Pyrroloquinoline quinone disodium levels (%) | | | |
| --- | --- | --- | --- | --- |
|  | 0 | 0.15 | 0.30 | 0.45 |
| Digestible energy^1^, MJ/kg | 14.57 | 14.49 | 14.60 | 14.55 |
| Dry matter | 88.60 | 87.92 | 88.41 | 88.83 |
| Crude protein | 20.23 | 20.36 | 20.41 | 20.38 |
| Lysine | 1.49 | 1.50 | 1.52 | 1.52 |
| Methionine | 0.45 | 0.46 | 0.46 | 0.46 |
| Threonine | 0.93 | 0.94 | 0.95 | 0.95 |
| Tryptophan | 0.27 | 0.26 | 0.27 | 0.27 |
| Calcium | 0.78 | 0.79 | 0.78 | 0.78 |
| Total phosphorus | 0.67 | 0.67 | 0.68 | 0.67 |

^1^Digestible energy are calculated values and other composition in the table are analyzed values.

**Supportal Table 3. Information of primary antibodies used for western blotting analysis**

| Antibody | Catalog no. | Source | Company | Producing area |
| --- | --- | --- | --- | --- |
| ZO-1 | #5406 | Rabbit | Cell Signaling Technology | Danvers, MA, USA |
| ZO-2 | #2874 | Rabbit | Cell Signaling Technology | Danvers, MA, USA |
| ZO-3 | ab205882 | Rabbit | Abcam | Cambridge, MA, USA |
| Occludin | 27260-1-AP | Rabbit | ProteinTech Group, | Chicago, IL, USA |
| Claudin-1 | 13050-1-AP | Rabbit | ProteinTech Group | Chicago, IL, USA |
| Bax | 50599-2-Ig | Rabbit | ProteinTech Group | Chicago, IL, USA |
| Bcl-2 | 12789-1-AP | Rabbit | ProteinTech Group | Chicago, IL, USA |
| Caspase-3 | 19677-1-AP | Rabbit | ProteinTech Group | Chicago, IL, USA |
| HO-1 | 10701-1-AP | Rabbit | ProteinTech Group | Chicago, IL, USA |
| Nrf2 | 16396-1-AP | Rabbit | ProteinTech Group | Chicago, IL, USA |
| Tubulin | #2125 | Rabbit | Cell Signaling Technology | Danvers, MA, USA |
| PCNA | #13110 | Rabbit | Cell Signaling Technology | Danvers, MA, USA |
| β-actin | #4970 | Rabbit | Cell Signaling Technology | Danvers, MA, USA |

**Supplemental Table 4. Primer sequences of target and reference genes**

|  | **Accession number** | **Primer sequences (5' to 3')** | **Product size (bp)** |
| --- | --- | --- | --- |
| β-actin | XM_021086047.1 | F CCACGAAACTACCTTCAACTC | 131 |
|  |  | R TGATCTCCTTCTGCATCCTGT |  |
| Caspase-3 | NM_214131.1 | F ACCCAAACTTTTCATAATTCA | 145 |
|  |  | R ACCAGGTGCTGTAGAATATGC |  |
| Bcl-2 | XM_021082883.1 | F AGAGCCGTTTCGTCCCTTTC | 270 |
|  |  | R GCACGTTTCCTAGCGAGCAT |  |
| Bax | XM_013998624.2 | F ATGATCGCAGCCGTGGACACG | 296 |
|  |  | R ACGAAGATGGTCACCGTCTGC |  |
| Nrf2 | XM_021075133.1 | F TTGTCTGTGATGCCAACGTG | 190 |
|  |  | R TTGGAACCGTGCTAGTCTCA |  |
| HO-1 | NM_001004027.1 | F AGGCTGAGAATGCCGAGTTC | 90 |
|  |  | R TGTGGTACAAGGACGCCATC |  |

F, forward primer; R, reverse primer.
